# Supplementary material for: Characterization of Humanized Mouse Model of Organophosphate Poisoning and Detection of Countermeasures via MALDI-MSI
Source: Int J Mol Sci. 2024 May 22;25(11):5624. doi: 10.3390/ijms25115624 (PMC11172367; doi:10.3390/ijms25115624)
Supplement: Supplementary file 1 [file ijms-25-05624-s001.zip › ijms-2981401-SI.pdf]

Supporting Information

Characterization of a Humanized Mouse Model of Organophosphate Poisoning and Detection of Countermeasures via MALDI-MSI

Caitlin M. Tressler[1*], Benjamin Wadsworth[2], Samantha Carriero[2], Natalie Dillman[1], Rachel Crawford[1], Tae-Hun Hahm[1], Kristine Glunde[1,4,5] and C. Linn Cadiuex[2]

[1]The Johns Hopkins University Applied Imaging Mass Spectrometry Core and Service Center; Division of Cancer Imaging Research; The Russell H. Morgan Department of Radiology and Radiological Science; The Johns Hopkins University School of Medicine; Baltimore; Maryland.

[2]United State Army Medical Research Institute for Chemical Defense

[4]The Sidney Kimmel Comprehensive Cancer Center, The Johns Hopkins University School of Medicine, Baltimore, Maryland, United States, 21205.

[5]Department of Biological Chemistry, The Johns Hopkins University School of Medicine, Baltimore, Maryland, United States, 21205.

*Corresponding author. Email: ctressl3@jh.edu Phone: (814) 931-1098 Fax: (410) 614-1948

Caitlin Tressler, Ph.D.
Assistant Professor of Radiology
Assistant Director, Applied Imaging Mass Spectrometry (AIMS)
The Johns Hopkins University School of Medicine
Russell H. Morgan Department of Radiology
and Radiological Science
Division of Cancer Imaging Research
720 Rutland Avenue
Traylor Building, Room 203
Baltimore, MD 21205
U.S.A.

Core website: www.hopkinsmedicine.org/radiology/aims

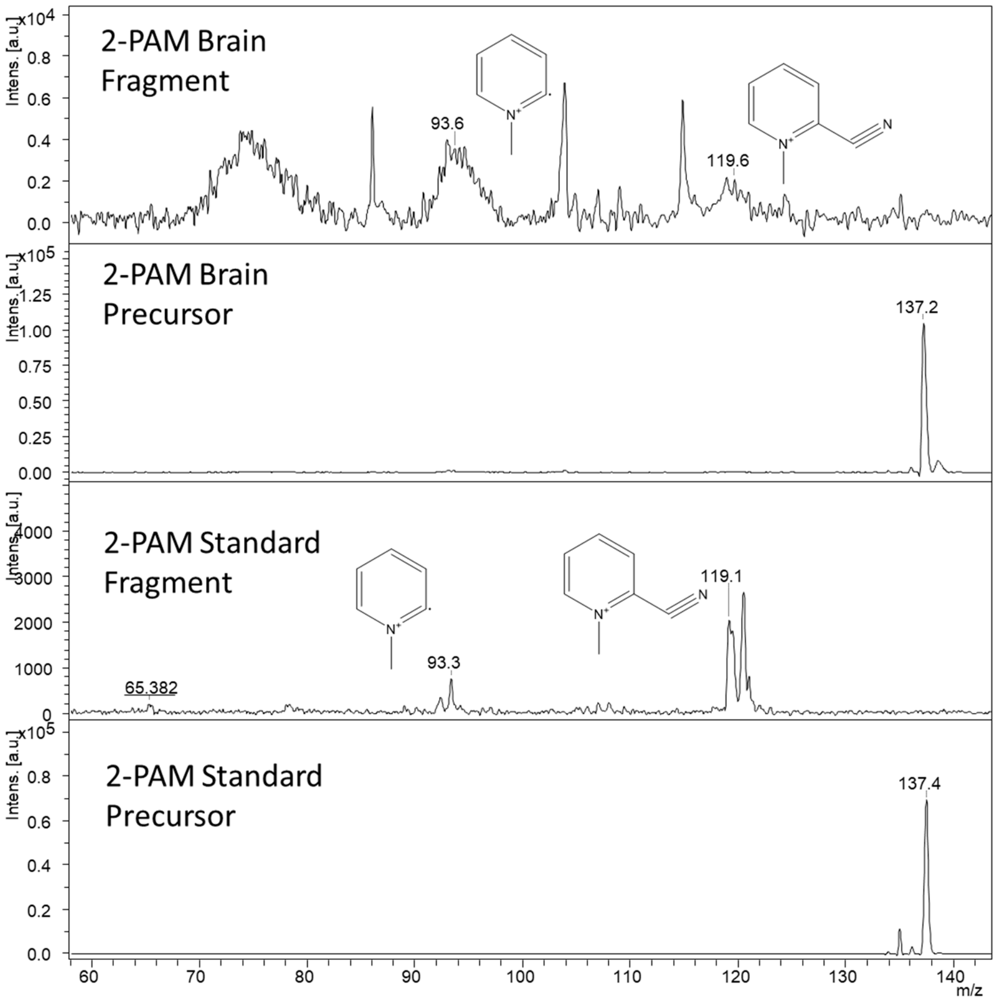

Figure S1. MSMS of m/z 137 (2PAM) in brain and standard

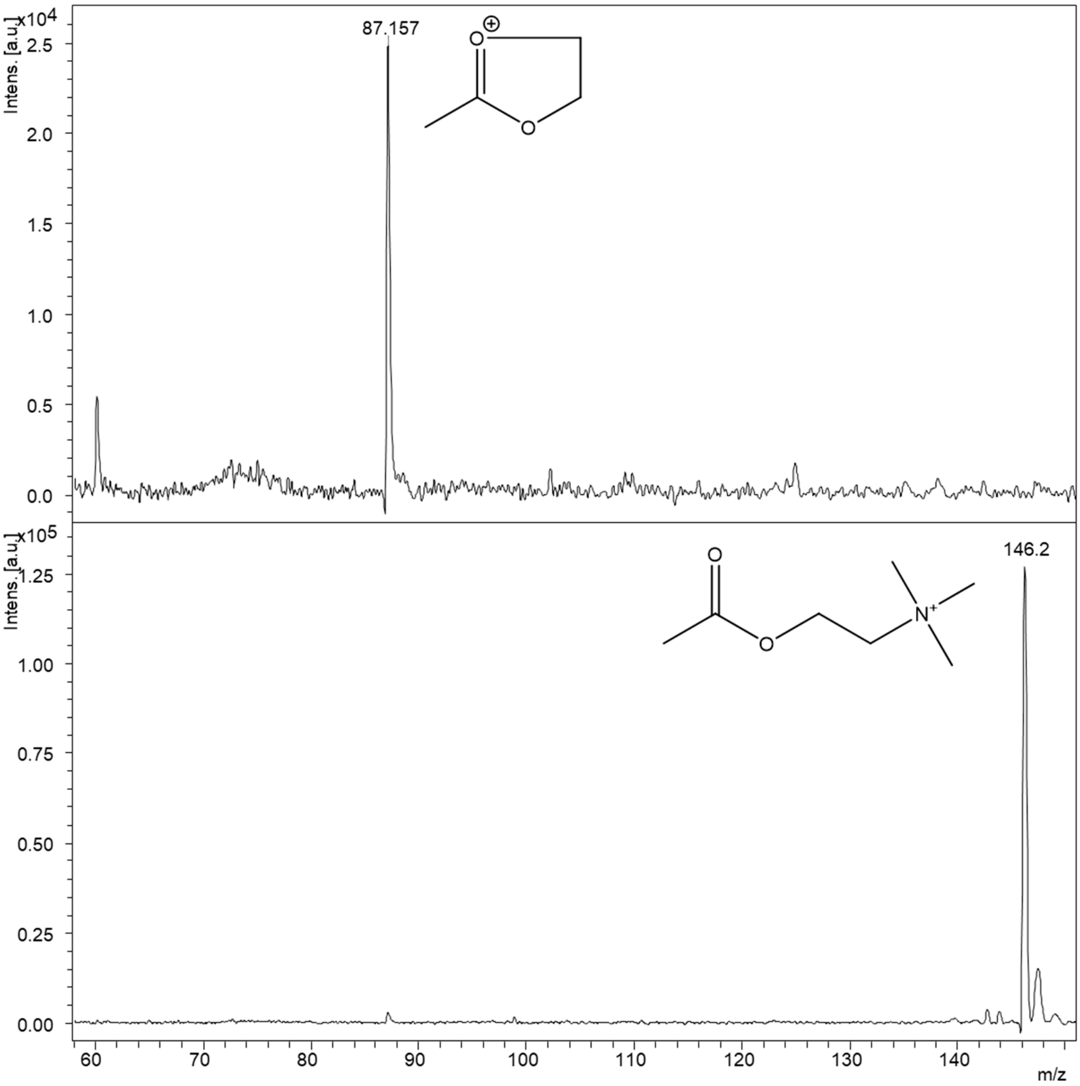

Figure S2. MSMS of m/z 146 (Ach).

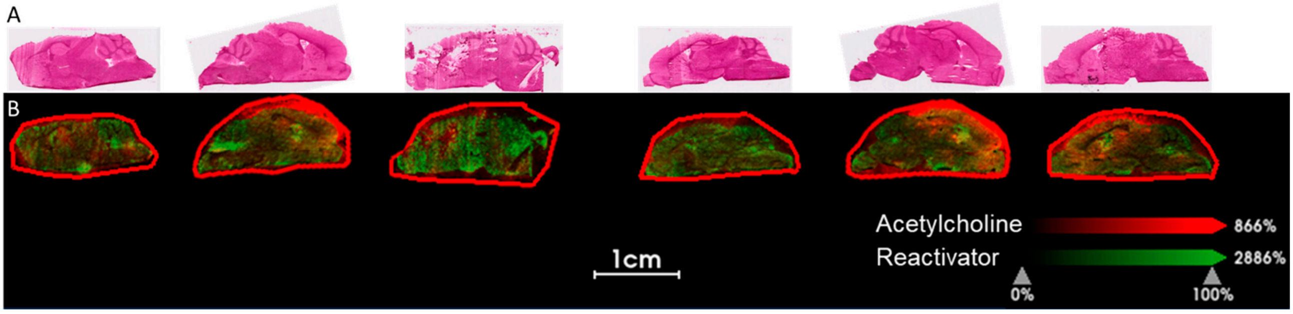

Figure S3. A. H&E stains of male (left) and female (right) brains. B. Overlays of Ach (red, m/z 146.1) and Reactivator (green, m/z 137.1) of fifteen-minute timepoint in OPNA and Reactivator treated brains with atropine for all biological replicates.

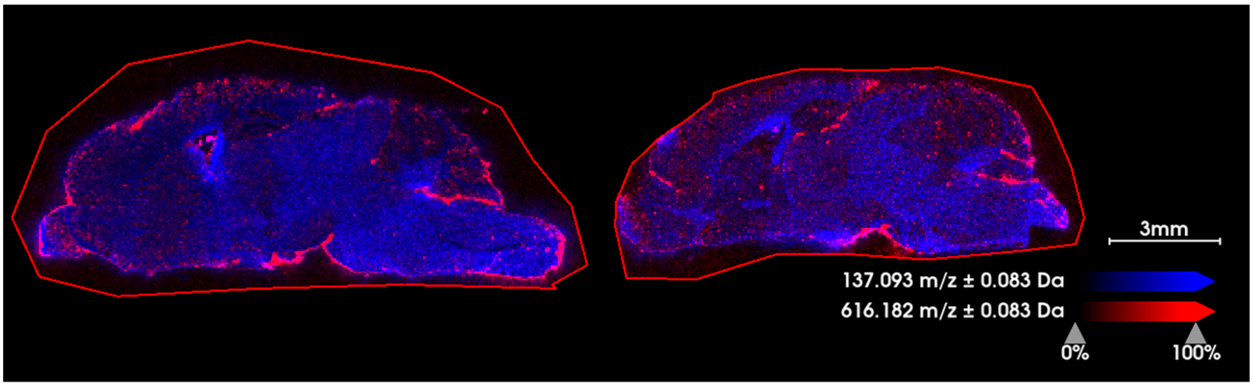

Figure S4. Overlay of 2-PAM (m/z 137.1, blue) and heme (m/z 616.2, red) in female (left) and male (right) KIKO mouse brains which have been dosed with OPNA and treated with Reactivator and atropine.
